# Efficacy of two diode lasers in the removal of calculus from the root surface: An in vitro study

**DOI:** 10.1002/cre2.769

**Published:** 2023-08-30

**Authors:** Domenico Marcattili, Leonardo Mancini, Francesco Tarallo, Fabio Casalena, Carla Pietropaoli, Enrico Marchetti

**Affiliations:** ^1^ Department of Life, Health & Environmental Sciences University of L'Aquila L'Aquila Italy

**Keywords:** debridement, dental calculus, diode laser, root planing, scaling

## Abstract

**Introduction:**

Scaling and root planning (SRP) is still the gold standard of nonsurgical periodontal therapy, and it has been accompanied by several supportive therapies in recent years. One of the most studied methods is the diode laser, thanks to its thermal and bactericidal properties. Our trial intended to verify whether it could influence the chemical bond between calculus and the root surface.

**Objectives:**

The aim of the study was to assess the efficacy of the diode laser prior the mechanical removal of calculus in an in vitro application. The reduction in time and the number of strokes required to clean the untreated root surfaces were evaluated as primary outcomes. The pressure was considered as a secondary outcome.

**Methods:**

A total of 75 extracted human teeth with subgingival calculus were assigned equally among three treatment groups (*n* = 25) according to the size of the occupied areas, which were classified by evaluating the pixel numbers. The groups were assigned to either no pretreatment application (A), Laser Diode Fox III (Sweden & Martina) (B) or Wiser Laser Evolution (Doctor Smile) (C). The weight for instrumentation was calibrated for an After Five curette (Hu‐Friedy, Chicago). A new set of tools was used for each group, and the curettes were sharpened after each use with the Sidekick sharpener (Hu‐Friedy, Chicago).

**Results:**

A Kruskal–Wallis test was used to assess the significance for each considered parameter. The results were statistically significant for each parameter for the two groups where the laser was used compared to the control group.

**Conclusions:**

Despite the limitations of an in vitro study, data showed that the diode laser had an overall positive effect on root debridement, facilitating SRP in terms of stroke count, time, and pressure.

## 1 INTRODUCTION

Periodontitis is a chronic inflammatory disease associated with a biofilm of dysbiotic bacteria that results in the progressive destruction of the tooth‐supporting apparatus. It is characterized by the loss of periodontal support tissue, manifested through gingival bleeding, clinical attachment loss (CAL), the presence of periodontal pockets, and the loss of alveolar bone, which is assessed radiographically (Papapanou et al., 2018). It is one of the main reasons for adult tooth loss (Jenkins et al., 1988) and needs adequate treatment.

Treating periodontal disease is a very complex challenge. Initially, it is essential to remove the biofilm from the root and subgingival surface (Van der Weijden & Timmerman, 2002). The main approach of nonsurgical periodontal therapy is scaling and root planning (SRP), which is used to remove tartar and subgingival biofilm. Subgingival calculus is one of the factors that contribute to the cyclical nature of periodontal disease (Moskow, 1970). The removal of tartar represents one of the greatest challenges in nonsurgical periodontal therapy, especially when it is found in harder areas to reach, such as the bottom of the periodontal pocket or the furcation of multi‐rooted teeth. Sherman et al. (1990a) analyzed the effectiveness of SRP by evaluating residual calculus and found the presence of calculus residues on 75% of teeth examined under a stereomicroscope, Allen and Kerr (1965) showed that the presence of calculus, even if sterile, caused a toxic effect on surrounding tissues, highlighting that the calculus must be completely removed to eliminate its inflammatory effect.

The role of calculus as an inflammatory factor is still under discussion. Sherman et al. showed that gingival changes after SRP, such as probing depth, bleeding, and periodontal attachment, are not affected by the presence of residual calculus, (Sherman et al., 1990b), although the formation of a new epithelial attachment has been observed in areas where residual calculus are present (Listgarten & Ellegaard, 1973).

In the last decade, several lasers have been used as a support in the treatment of periodontitis; among the most used is the diode laser (DL) (809–980 nm), with the Nd:YAG being capable of both decontaminating and remodeling the tissues (Cobb et al., 2010). Moreover, the laser increases hemostasis through coagulation and occlusion of arterioles, venules, and capillaries, induced by heat, (Cobb et al., 2010), allowing a visible and clean operating field. The heat given off by the laser also has a bactericidal effect on the target site (Cobb et al., 2010).

Considering the microbial components, laser irradiation, with its bactericidal effect, could represent a valid aid to “nonsurgical periodontal therapy” (SRP) (Moritz et al., 1998). As already mentioned, all lasers have a thermal effect, and the non‐spore‐producing bacteria, including the anaerobic bacteria responsible for periodontal disease, are deactivated at a temperature of around 50°C (Cobb, 2006; McDavid et al., 2001). In addition, coagulation of the inflammatory tissue of the periodontal pocket and hemostasis are also obtained at 60°C. A further effect to be considered in nonsurgical periodontal therapy is the possibility of the DL weakening the chemical bond that allows the adhesion of calcified deposits to the root surface, through a photochemical effect, thus facilitating their removal with conventional instruments (Ben Hatit et al., 1996; Chantaboury & Trinakis, 2005; Cobb, 1996; Midda, 1992). The purpose of this study was to evaluate whether using the DL in the pretreatment SRP facilitates the removal of tartar deposits through the weakening of the bond between calculus and the root surface.

## 2 MATERIALS AND METHODS

SRP was performed using manual instruments on the dental surfaces with the presence of calculus after undergoing three different pretreatment procedures. The aim of the study was to evaluate the number of strokes and the time required to remove the calculus from the root surface, to evaluate the possible positive effect of the laser used before the nonsurgical periodontal therapy.

### 2.1 Sample preparation and distribution

Human teeth extracted for periodontal reasons were stored in a saline solution (NaCl 0.90% w/v) and later assigned to one of the three treatments. The collection of human teeth and their use for study purposes was approved by the Internal Review Board of the University of Study of L'Aquila (No.: 02/2019). All teeth showed tartar under the enamel‐cement junction, occupying variable areas in terms of size, and position along the root. All 75 teeth satisfied the main inclusion criteria: intact and caries‐free root surfaces displayed plain root areas affected by calculus. Single and multirooted teeth were equally included.

Each sample was positioned horizontally in a block of resin (Palapress) to provide fixation during debridement and standardized positioning for photographic analysis. The samples were bi‐digitally fixed with the exposed calculus surface on the top to allow direct access for the treating agents and curettes. All samples were consecutively numbered from 1 to 75.

**Figure 1:**
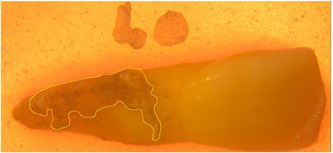
Labeling the area of interest (yellow line) and its extension upon a plane portion of the root.

All sites of interest were photographed (7200D; Nikon) under a stereomicroscope (Leica). The areas covered with calculus were manually determined and measured by calculating the number of pixels (Preibisch et al., 2009). To account for the equal distribution of areas affected by calculus among the three treatment groups, the assignment of the total number of teeth (*n* = 75) to each group was stratified by the pixel count (Figure 1).

### 2.2 Calculus pretreatment in SRP

**Figure 2:**
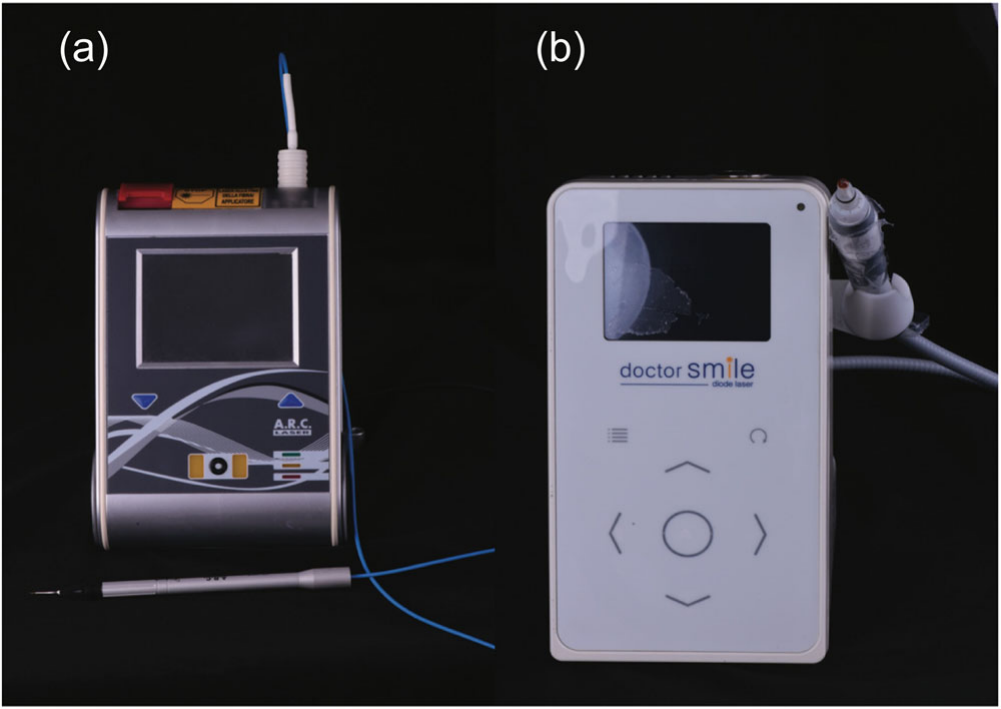
(a) Laser diode Fox III (Sweden & Martina). (b) Wiser laser evolution (Doctor Smile).

**Figure 3:**
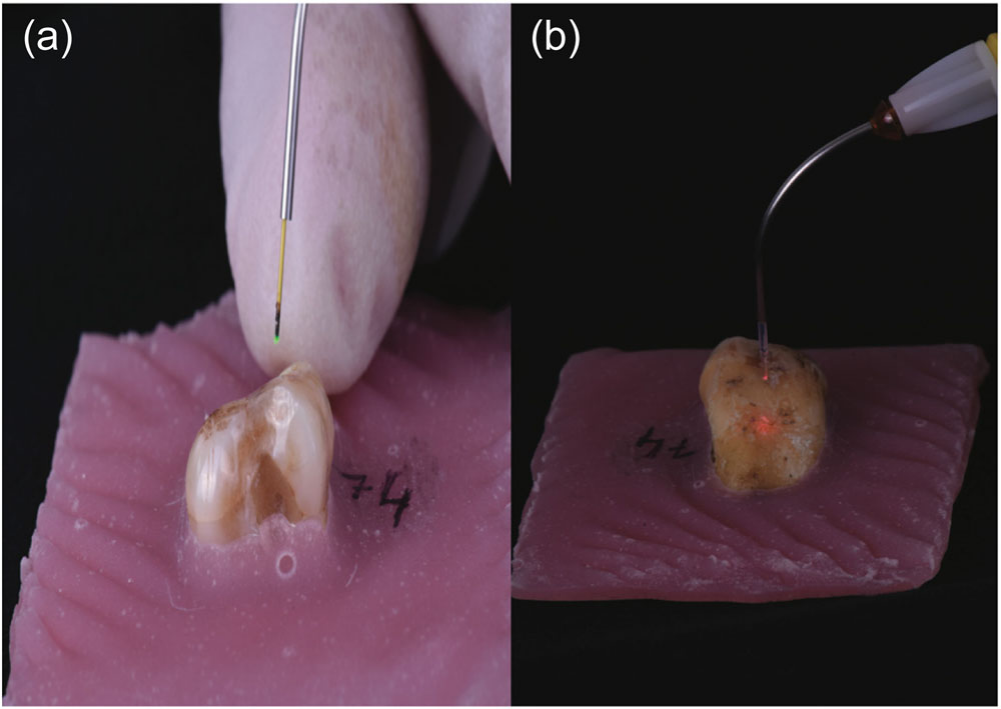
(a) Laser diode Fox III (Sweden & Martina) tip activated. (b) Wiser laser evolution (Doctor Smile) tip activated.

The samples of Group B were pretreated with Fox III DL (Sweden & Martina) with a fiber of 300 µm, a power of 2.5 W, 10 ms pulse length, and a 10 ms pulse pause for 30 s (Figures 2a and 3a). The samples of Group C were pretreated with Wiser Laser Evolution (Doctor Smile) with a frequency of 2.5 W ‐ average 0.7 W, pulse length of 30 ms, and pulse pause 70 ms for 30 s (Figures 2b and 3b). Both DLs were used according to the manufacturer's indications. No additional treatment to SRP was applied in the control group (group A). Each group was supplied with a new curette (After Five, Hu‐Friedy, Chicago); working strokes were performed from apical to coronal, parallel to the axis of the tooth. A weight of 800–1000 g was applied during scaling, and the instrument was sharpened after each use (Sidekick, Hu Friedy, Chicago) (Busslinger et al., 2001).

**Figure 4:**
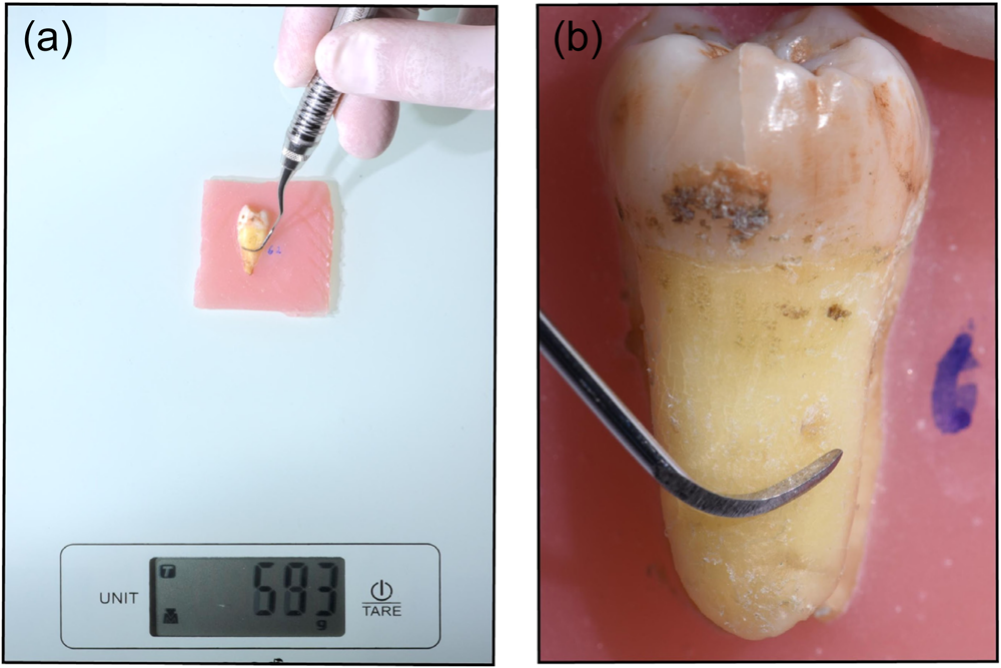
(a) Strokes on the balance. (b) Detail of strokes on the tooth.

The first investigator (MB) selected and preconditioned the specimens. The second, blinded, investigator (CP) carried out all treatments, recorded the time required for treatment (in seconds), as well as the number of strokes, monitored the weight applied during treatment, and proved the thoroughness of the debridement by the tactile detection of remnant calculus on the surfaces (Figure 4a,b).

Each sample was treated until the area of interest showed no residual calculus by visual and tactile inspection (CP‐15 Probe, Hu‐Friedy, Chicago). After root debridement, the surfaces were photographed under microscopic magnification to record the results.

### 2.3 Statistical analysis

**Figure 5:**
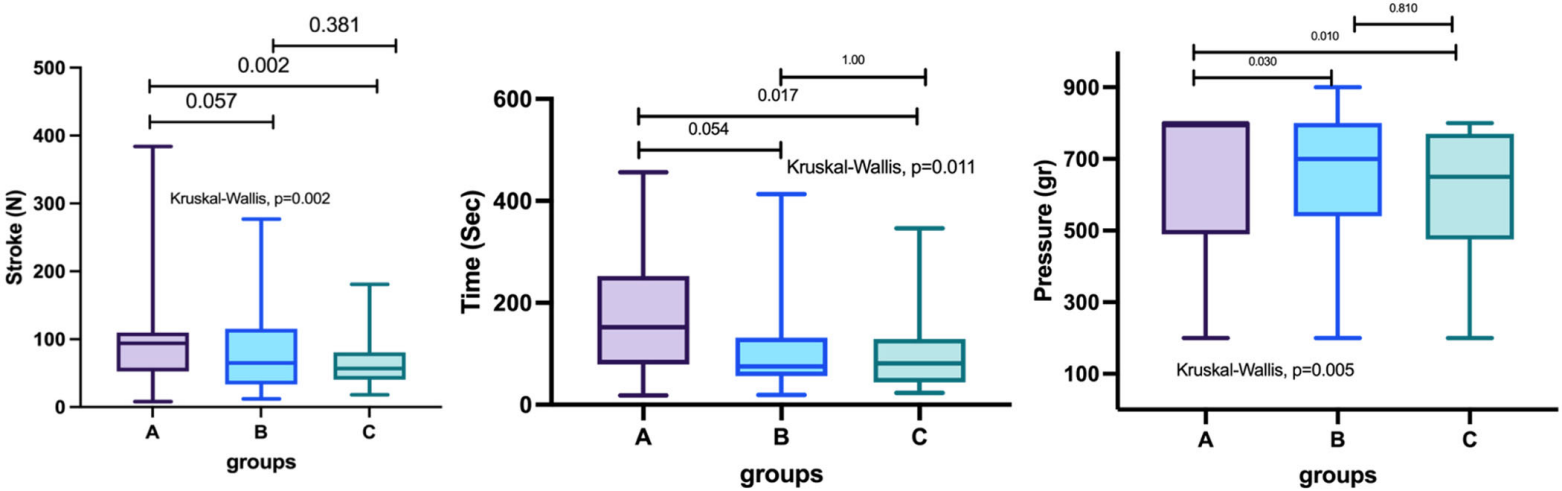
Plots with the results analyzed by the Kruskal–Wallis test. In each group (pressure, time, and strokes), statistically significant results between the test groups (b and c) and the control group (a) were recorded.

Data were recorded in a spreadsheet and analyzed by a non‐parametric approach because of the non‐normal distribution checked using Kolmogorov–Smirnov's test. Specifically, the overall p‐trend for each outcome of interest (i.e., pressure, time and numbers of strokes) was explored by the Kruskal–Wallis test (Figure 5), while pairwise comparisons were evaluated by the Mann–Whitney's test with Bonferroni's correction. Medians and interquartile ranges (IQR) were derived and presented in the results (Table 1). Data were analyzed as recorded, and no missing data were present. The level of statistical significance was set at *p* > .05. Post hoc power was estimated at 77% to detect a large effect size (*d* = 0.8) at the difference of a parameter between two groups.

**Table 1:** Median and interquartile range (IQR) of the time, strokes, and pressure of the three groups.

	Group	*N*	Median	IQR	*p*‐Value (KW)	*p*‐Value (MW)
Time (s)	A	25	175	98–282	.011	A versus B; *p* = .054 A versus C; *p* = .017 B versus C; *p* = 1.000
	B	25	75	57–126		
	C	25	81	44–128		
Pressure (gr)	A	25	920	575–920	.005	A versus B; *p* = .030 A versus C; *p* = .010 B versus C; *p* = .810
	B	25	700	550–800		
	C	25	650	500–750		
Strokes (N)	A	25	30	25–35	.002	A versus B; *p* = .057 A versus C; *p* = .002 B versus C; *p* = .381
	B	25	24	15–33		
	C	25	16	8–26		

*Note*: Kruskall–Wallis (KW) test was used for differences at distributions between the three groups. Mann–Whitney (MW) test was adjusted with Bonferroni for the pairwise comparisons.

## 3 RESULTS

The average tartar present on each tooth, expressed in pixels, was 1,637,705 ± 1,617,704 pixels in Group A (control group), 1,536,570 ± 1,392,091 pixels in Group B (Laser Diode Fox, Sweden & Martina), and 1,420,084 ± 1,014,378 pixels in Group C (Wiser Laser Evolution, Doctor Smile).

The average tartar expressed as a percentage of the total tooth surface was 17.53 ± 0.13% in Group A (control group), 16.92 ± 0.12% in Group B (Laser Diode Fox, Sweden & Martina), and 17.40 ± 0.13% in Group C (Wiser Laser Evolution, Doctor Smile).

There were no statistically significant differences in the homogeneity of the three groups, both as a function of the average tartar present on each tooth and of the average tartar expressed as a percentage of the total surface of the tooth.

### 3.1 Time

The time, expressed in seconds, for each group was as follows (median, IQR): Group A (control group) 175 (98–282) s, Group B (Group, Laser Diode Fox, Sweden & Martina) 75 (57–126) s, and Group C (Group, Wiser Laser Evolution, Doctor Smile) 81 (44–128) s.

There were statistically significant differences among the three groups (*p* = .011), but not between Group B (Laser Diode Fox, Sweden & Martina) and Group C (Wiser Laser Evolution, Doctor Smile) (*p* = 1.000). There were statistically strong differences between Group A (control group) and Group B (Laser Diode Fox, Sweden & Martina) (*p* = .054) and significant between Group A (control group) and Group C (Wiser Laser Evolution, Doctor Smile) (*p* = .017) (Table 1).

### 3.2 Pressure

The pressure, expressed in bar, for each group was as follows (median, IQR): Group A (control group) 920 (575–920) bar, Group B (Laser Diode Fox, Sweden & Martina) 700 (550–800) bar, and Group C (Wiser Laser Evolution, Doctor Smile) 650 (500–750) bar.

There were statistically significant differences among the three groups (*p* = .005), except between Group B (Laser Diode Fox, Sweden & Martina) and Group C (Wiser Laser Evolution, Doctor Smile) (*p* = .810). There were statistically significant differences between Group A (control group) and Group B (Laser Diode Fox, Sweden & Martina) (*p* = .030), and between Group A (control group) and Group C (Wiser Laser Evolution, Doctor Smile) (*p* = .010) (Table 1).

### 3.3 Strokes count

The number of strokes was 30 (25–35) (median, IQR) in Group A (control group), 24 (15–33) in Group B (Laser Diode Fox, Sweden & Martina), and 16 (8–26) in Group C (Wiser Laser Evolution, Doctor Smile).

There were statistically significant differences among the three groups (*p* = .002), but not between Group B (Laser Diode Fox, Sweden & Martina) and Group C (Wiser Laser Evolution, Doctor Smile) (*p* = .381). There were statistically strong differences between Group A (control group) and Group B (Laser Diode Fox, Sweden & Martina) (*p* = .057), and between Group A (control group) and Group C (Wiser Laser Evolution, Doctor Smile) (*p* = .002) (Table 1).

In the following figure, *p*‐values should be updated:

## 4 DISCUSSION

Root surface debridement is considered the fundamental and necessary practice to decontaminate the root surface, eliminate the bacterial component and promote healing with subsequent clinical attachment improvement (Arcuri et al., 2020; Sanz et al., 2012). However, numerous studies in the literature have proposed alternative methods to achieve the identical goal (Katsikanis et al., 2020; Sumra et al., 2019). Despite numerous efforts and several of both chemical and mechanical technologies, SRP still proved to be the gold standard (Katsikanis et al., 2020; Sanz et al., 2012). In fact, the efforts proposed do not seem to gain higher clinical results in treating periodontitis for a lengthy term. Reasonably, these techniques have been considered useful as a coadjutant in support to SRP. The laser is sort of a strategy.

Several advantages of the laser include extreme compactness, affordability, ease of operation, simple setup, and versatility. DL is particularly useful in periodontal treatment because its wavelengths are highly absorbed in the melanin and hemoglobin found in the soft tissue. When patients are affected by periodontitis, biofilm communities tend to increase. Healthy pink tissue becomes red in color (because it has more pigment) and is associated with increased bleeding due to inflammation (Verma et al., 2012). For this reason, we chose to compare the effectiveness of two DLs despite the fact that, since this is an in vitro study, there are no inflamed soft tissues in which the laser performs the aforementioned functions.

Therefore, the main objective of this in vitro study was to ascertain whether the use of the DL could facilitate SRP treatment by decreasing the patient's chair time and the number of applications of both manual and mechanical instruments. The results of this trial suggest that there may be a development in accomplishing whole root debridement of calculus through SRP preceded by the usage of a DL. Statistically significant variations were determined among all three groups in terms of time, pressure, and the number of strokes required to achieve the elimination of calculus. The distribution of the expanse of the tooth surface covered by calculus was made uniform by the stratified distribution within the three treatment groups. The specific reason why these results were obtained is still unclear but appears to be due to a weakening of the chemical bond between the hematic tartar and the root surface. In fact, other studies (Chantaboury & Trinakis, 2005; Cobb, 1996; Midda, 1992) agree that the use of the laser may alter the chemical bond between tartar and root surface through the application of an energy source. A plausible explanation could be that the tissue absorbs an amount of radiation per volume and transforms it into an amount of energy, depending on the exposure time used. This speculation can also be supported by the fact that both lasers used were pulsed light. According to numerous studies, a pulsed‐light‐laser can emit power peaks significantly greater than their average power. Therefore, pulsed lasers offer very efficient spot‐welding capability, which proves to be an advantage, especially where greater penetration through the tissues is required (Assuncao & Williams, 2013).

Moreover, the results of our study are also supported by Roncati et al., in which the DL was used to pretreat the root surface to facilitate its subsequent removal with mechanical instrumentation (Roncati & Gariffo, 2014). To the best of our knowledge, other studies in the literature that have used the DL to precondition the root surface, obtaining results contrasting with those demonstrated in our study, have not been reported. Instead, contrasting results were reported by Becker et al., where the preconditioning of calculus present on the root surface with an alkaline solution based on hypochlorite and amino acids did not show statistically significant results in terms of instrumentation time and number of strokes performed by curettes (Becker et al., 2018). The authors asserted that the solution did not chemically alter the surface of the tartar, in fact, not increasing the effectiveness of the SRP. It is plausible that the conflicting results obtained in their study are due to an insufficient exposure of root calculus to the chemical agent making its use ineffective.

Furthermore, the following limitations ought to be considered: the low sample size which may also jeopardize the results, the in vitro model, which may simplify the clinical reality (no interference of blood) and the bidimensional analysis of the extension of calculus on the root surface while it should be taken into account in its three‐dimensionality for a more reliable reading. This could have led to a nonhomogeneous distribution within the three groups under consideration since only the width of the tartar and not the thickness has been considered.

## 5 CONCLUSION

The results of the study showed that the use of the diode laser before SRP is associated with a statistically significant reduction of time, pressure, and strokes when compared to the control group. These advantages may bring to multiple benefits for the patient in terms of time preserving and pain relief. Further in vivo studies, which may also include patients' related outcomes, are suggested to better clarify the role of diode laser in preconditioning root tartar and therefore simplify the SRP procedure.

## SCIENTIFIC RATIONALE

The removal of calculus from the root surface is still the most challenging goal in periodontitis therapy to promote soft tissue healing. Preconditioning the tartar with the diode laser appears to be a valid method to facilitate its removal.

## PRINCIPAL FINDINGS

The use of diode laser before SRP demonstrated a decrease in time, curette strokes count and force intensity required for root calculus removal.

## PRACTICAL IMPLICATIONS

The use of diode laser as preconditioner of root surface calculus before SRP may decrease patients' chair time and obtain pain relieving.

## AUTHOR CONTRIBUTIONS

Enrico Marchetti, Domenico Marcattili, and Fabio Casalena: Methodology. Carla Pietropaoli and Leonardo Mancini: Validation. Domenico Marcattili and Leonardo Mancini: Investigation. Enrico Marchetti: Resources. Leonardo Mancini, Francesco Tarallo, and Domenico Marcattili: Writing original draft preparation. Fabio Casalena: Statistical analysis. Francesco Tarallo and Leonardo Mancini: Writing review and editing. Francesco Tarallo and Enrico Marchetti: Supervision. Enrico Marchetti and Carla Pietropaoli: Project administration. All authors have read and agreed to the published version of the manuscript.

## CONFLICT OF INTEREST STATEMENT

No potential conflict of interest relevant to this article was reported. Only Enrico Marchetti has had a collaborative relationship with the Sweden & Martina company from 2017 in a different field from that of the study.

## Data Availability

The data that support the findings of this study are available on request from the corresponding author. The data are not publicly available due to privacy or ethical restrictions.
